# Spiritual Care and Spiritual Perspective: Assessing Oncology Patients’ Perspectives and Their Implications for Healthcare Management

**DOI:** 10.3390/healthcare13131554

**Published:** 2025-06-29

**Authors:** Monica Elisa Meneses-La-Riva, Víctor Hugo Fernández-Bedoya, Josefina Amanda Suyo-Vega, Hitler Giovanni Ocupa-Cabrera, Giovanni di Deus Ocupa-Meneses

**Affiliations:** Grupo de Investigación Innovación Humanizadora, Universidad César Vallejo, Av. Alfredo Mendiola, Lima 15311, Peru; jsuyov1@ucv.edu.pe (J.A.S.-V.); hocupa@ucvvirtual.edu.pe (H.G.O.-C.); giovanniocupa56480@gmail.com (G.d.D.O.-M.)

**Keywords:** spirituality, spiritual care, quality of care, service quality, hospital, spiritual perspective, nursing care

## Abstract

Background: Spiritual care is vital for the holistic well-being of hospitalized cancer patients, addressing their emotional, psychological, and spiritual needs. This study addresses gaps in the relevant literature by evaluating spiritual perspectives among Peruvian oncology patients, offering culturally grounded insights that can inform nursing practice and healthcare management. The main objective of this research was to measure the overall level of Spiritual Perspective among hospitalized oncology patients using the Spiritual Perspective Scale (SPS) developed by Pamela Reed in 1987, which reflects early aspects of spirituality later integrated into her broader Spiritual Perspective theory. Materials and methods: This study aimed to evaluate the perceived levels of Spiritual Perspective among oncology patients in a hospital setting. Adopting a quantitative, descriptive, cross-sectional design, data were gathered from 137 patients at a national hospital in Lima, Peru. Results: The majority of participants were older adults, with a high school education, and predominantly single. Findings revealed that most patients experienced moderate levels of Spiritual Perspective, spiritual practices, and beliefs. Patients commonly practiced prayer, meditation, and spiritual reading. Beliefs centered on a higher power and forgiveness. Essential support networks also provided emotional aid, complementing spiritual care. Conclusions: This study highlights the importance of spiritual care in nursing for oncology patients. Findings advance understanding of spirituality in illness and support interventions to improve patient outcomes.

## 1. Introduction

Currently, reports from the World Health Organization (WHO) highlight a global increase in oncological diseases. This development has transformed cancer into an unprecedented public health challenge, given its alarming mortality and morbidity statistics. Within this context, the disease demands significant attention in terms of healthcare services, the need for well-trained professionals, and the provision of highly specialized care [1].

In the context of specialized nursing care, it is imperative to offer cancer patients palliative care that incorporates spirituality. Spirituality represents a facet of inner life, often underexplored and underserved by healthcare professionals. Nevertheless, it is a dimension that individuals relentlessly seek to attain a sense of balance in their health. When this equilibrium is disrupted, it triggers the body’s defense mechanisms, all aimed at safeguarding and preserving inner peace [2].

In this regard, the nursing professional takes on the role of accompanying individuals who are suffering, assuming the responsibility of satisfying their basic needs, providing comfort, and alleviating their suffering throughout the course of their illness. Moreover, it is undeniable that spiritual care has a profound impact on the quality of life and the patient’s perspective on illness and mortality [3,4]. Intellectual humility strengthens critical thinking, aiding patients in reflection, evaluation, and decision-making [5].

On the other hand, the expressions and spiritual practices adopted by patients are events that foster inner strength and imbue human existence with meaning [6]. Hence, individuals who fall ill or experience the loss of a family member require this inner energy to safeguard their personal well-being. Moreover, the healthcare professionals responsible for the patient’s care must also address their spiritual needs [7]. To enhance the quality of life and emotional well-being, there is a need for an emphasis on interrelation and humanized care, which aims to fulfill spiritual and emotional needs 7. These essential emotional supports serve to bolster patient and family care [8,9].

Humanization involves commitment, dedication, and a shift away from a traditional paradigm toward a more humane approach [10,11,12,13]. Attributes that foster sensitivity [14], creativity, the cultivation of values, and spirituality collectively highlight the qualifications, identity, and vocation of nursing professionals. Hence, it is imperative to incorporate into the curriculum the education and training of expert professionals in spiritual care [15,16]. These professionals will manifest, in their practice, qualities such as humane treatment, religious understanding, empathy, compassion, and consolation, especially during the most crucial moments in life, marked by illness or the loss of a loved one. Such an approach views these experiences as opportunities for effective and efficient personal growth [17,18]. Furthermore, active listening and the expression of comfort through a compassionate embrace provide solace in times of human loss. Even going as far as bending the rules to allow loved ones to approach restricted areas to bid farewell to their dear ones is a fundamental aspect of a dignified end-of-life experience [6,14,19,20].

Similarly, within the realm of nursing care practice, there is often a lack of understanding regarding experiences such as meditation, reflection, and prayer. These practices hold significance in elevating spirituality and constitute forms of religious therapy. They contribute to the appreciation and intervention in the emotional dimension of individuals, recognizing them as holistic beings where there exists a harmonious unity of mind, body, and spirit [21,22,23,24].

On the other hand, spirituality, as an essential element of the human being, “is akin to a musical symphony of love” that accompanies the individual who is unwell [25,26,27]. This person feels, thinks, suffers, and questions the multitude of diagnoses that can overshadow the essence of their own life [28]. Particularly, concerning the enhanced management of stress and the religious/spiritual support offered by professionals, it is crucial that such support is provided promptly and with a positive attitude. This approach serves to bolster spiritual competencies through the respectful, responsible, compassionate, and affectionate interactions of nurses with their patients [29].

Spiritual care encompasses addressing the essential triad of body, mind, and spirit, which stands as a pivotal element in fortifying self-esteem and navigating the challenges of diagnosis, treatment, recovery, and even death [30,31,32]. Patients often engage in religious practices like prayer, reading the Bible, or singing praises as part of their routine. Additionally, various forms of spiritual support, such as meditation, contemplation of life’s purpose, connecting with nature, and listening to music, are regarded as sources of spiritual strength. These practices extend beyond emotional well-being and can alleviate the physical symptoms of the illness [33].

In other words, the profound connection to faith serves as a foundational support principle for the vulnerable population affected by cancer. It equips them to navigate the challenging aspects of the disease with greater resilience. Those who exhibit a stronger religious-spiritual coping mechanism tend to do so with a sense of hope. This hope acts as a strategy for confronting fear, uncertainty, and the stigma associated with suffering. These factors often bring about feelings of anxiety, depression, and, in some cases, even suicidal thoughts [34].

The theorist Pamela Reed highlights that spirituality includes the pursuit of meaning and existential purpose, establishing connections beyond the individual, cultivating ethical principles, practicing meditation and mindfulness, exploring nature’s interconnectedness, expressing oneself through art, surrendering to others, and seeking inner harmony [35,36]. These aspects are vital to consider, given that spirituality is a broad and subjective concept with significant variations among individuals.

Furthermore, spirituality is characterized as an inherent aspect of the human condition, enabling individuals to find meaning through a sense of connection with a supreme being or a higher purpose entity that transcends the individual self. This is fundamentally a human experience that empowers individuals to discover the significance and purpose of their lives, particularly when facing illness. These practices, experiences, and beliefs are rooted in the connection with a transcendent being, especially during stages and moments when the fragile awareness of mortality becomes apparent [37].

From the perspective of Pamela Reed’s humanistic care, two dimensions are established: spiritual practices, where each person holds unique beliefs and engages in specific spiritual activities, which have the capacity to evolve and change over time [35,37], and spiritual beliefs, which provide a sense of connection with a supreme entity or a being beyond the scope of human experience [35,37]. Likewise, prayer provides an atmosphere of peace, inner strength, and elevates the sense of hope [38,39].

Within spirituality, practices and beliefs are open to transformation and the integration of elements from various religions, mystical traditions, or esoteric currents, all of which aim to establish a connection with a higher being beyond oneself. Individuals develop unique life trajectories centered around their personal spiritual reality, where the extraordinary and the attainable merge to construct a narrative that serves as a guiding path towards the process of recovery and emotional well-being in everyday life [40].

Considering the aforementioned, it is emphasized that Spiritual Perspective is an integral dimension that should be approached from the standpoint of spirituality and religiosity. These aspects are inherent attributes of the human essence and encompass the entirety of the individual [41]. This approach is regarded as a positive way to cope with illness. Therefore, humanized care enhances both transpersonal and interpersonal treatment, enriching the nurse-patient relationship, especially in critical situations [42,43]. The bonds of trust and security enable the discussion of topics related to spirituality and religiosity, facilitating the anticipation of the patient’s desired rituals in end-of-life situations [44], because they favor the fullness of care and improve spiritual well-being [45,46]. Therefore, the overarching problem emerges: What is the overall level of Spiritual Perspective perceived by hospitalized oncology patients, according to Reed’s theoretical framework? What is the frequency of spiritual practices and the strength of spiritual beliefs among hospitalized oncology patients? Are there patterns or differences in perceived Spiritual Perspective based on the sociodemographic characteristics of hospitalized oncology patients?

While spiritual care is a broad and multidimensional concept that includes emotional presence, compassionate communication, and therapeutic relationships, this study focuses specifically on spiritual beliefs and practices as measurable components. These elements, drawn from Pamela Reed’s Spiritual Perspective Theory, represent core expressions of spirituality that are accessible to quantitative assessment and reflect key aspects of patients’ inner lives. Future studies could expand on this approach by integrating interpersonal or relational aspects of spiritual care more deeply.

### Theoretical Foundation and Research Objective

Pamela Reed’s theoretical perspective on Spiritual Perspective provides a robust framework for understanding the spiritual dimension of human existence [47]. According to Reed, Spiritual Perspective encompasses the pursuit of meaning, connection with entities beyond the self, and cultivation of ethical and moral principles. By grounding this research in Reed’s theory, we align with a comprehensive understanding of spirituality that extends beyond religious affiliations and encompasses a broad spectrum of human experiences.

In practical terms, addressing the spiritual needs of hospitalized cancer patients is essential for providing holistic care. Cancer diagnosis and treatment can evoke profound existential questions and spiritual distress in patients. By measuring the level of Spiritual Perspective perceived by patients in a hospital setting, we can tailor nursing interventions to meet their spiritual needs effectively. This research informs healthcare practices by highlighting the importance of integrating spiritual care into patient-centered approaches, ultimately improving patient outcomes and enhancing their overall well-being during the cancer care journey.

Despite increasing global attention to spiritual care in oncology, limited empirical evidence exists on how Spiritual Perspective is experienced by cancer patients in Latin America, particularly in Peru. Additionally, few studies have applied Pamela Reed’s theory quantitatively in hospital settings. This study addresses these gaps by evaluating Spiritual Perspective among Peruvian oncology patients, offering culturally grounded insights that can inform nursing practice and healthcare management.

Based on this rationale, the objectives of this study were as follows:

1. To measure the overall level of Spiritual Perspective among hospitalized oncology patients using the Spiritual Perspective Scale (SPS) developed by Pamela Reed in 1987, which reflects early aspects of spirituality later integrated into her broader Spiritual Perspective theory.
2. To assess the frequency of spiritual practices and the strength of spiritual beliefs.
3. To empirically validate the hypothesized two-factor structure of the scale, through confirmatory factor analysis.
4. To identify patterns in Spiritual Perspective based on sociodemographic characteristics.

While Reed’s theory emerged from a Western context emphasizing individual growth and personal spirituality, its dimensions also resonate with collectivist cultures like those in Latin America. In this context, Spiritual Perspective is often experienced through interpersonal relationships, family cohesion, and shared faith practices. Therefore, the application of Reed’s framework in this study was culturally adapted to highlight collective spiritual expressions, such as community prayer, family support, and religious traditions grounded in Peruvian society.

In the Peruvian context, evidence has been reported regarding the use of the Self-Transcendence Scale in oncology and chronic care settings. In one study, the relationship between self-transcendence, anxiety, and depression in cancer patients undergoing treatment was examined using Reed’s theoretical approach [48]. Additionally, the model has been applied in research on older adults with chronic non-communicable diseases in the southern region of the country, showing its relevance in geriatric nursing and long-term care [49]. These applications support the cultural adaptability of Reed’s theory and its utility in holistic nursing interventions within Peru.

Therefore, the application of Reed’s framework in this study was culturally adapted to highlight collective spiritual expressions, such as community prayer, family support, and religious traditions grounded in Peruvian society [50].

## 2. Methodology

To conduct this study, the STROBE statement with the checklist of elements to be included in reports of cross-sectional studies was adhered to [51].

### 2.1. Design and Participants

This study utilized a quantitative, descriptive, cross-sectional, and non-experimental design approach. It was conducted at a national hospital located in Lima, which offers care for various cancer-related specialties. It included the entire accessible population of hospitalized oncology patients within the participating department during the data collection period. This comprised 137 eligible patients who met the inclusion criteria. Therefore, a census of this specific departmental population was conducted. Data collection took place between January and May 2023. Each respondent was informed that data collection was solely for research purposes and the continuous improvement of service quality.

### 2.2. Data Collection Instruments

The Spiritual Perspective Scale (SPS), originally developed by Pamela Reed in 1987 to assess spiritual practices and beliefs, was used in this study [47]. Although this instrument predates the formal Spiritual Perspective Theory published by Reed in 1991 [47], it captures key dimensions that were foundational to the later theory, translated into Spanish by [35,37] which consists of 2 dimensions, spiritual practices and beliefs, with 10 items and a Likert scale with a criterion of 6 points to specify the level of spirituality of cancer patients, categorized into ranges: low (10–19), moderate (20–46), and high (47–60). In the dimensions: spiritual practices, the levels were considered as low (4–8), moderate (9–18), and high (19–24); and in spiritual beliefs, the levels were categorized as low (6–9), moderate (10–18), and high (19–36). This instrument is presented in Table 1, has a Cronbach’s alpha validity of 0.70 (moderately reliable), and was previously validated for use in Latin American contexts [35,37]. Prior to this study, the Spanish version underwent expert review by Peruvian nursing faculty and a pilot test with 15 patients to ensure semantic and cultural relevance. Minor linguistic adjustments were made to adapt terms to Peruvian Spanish while preserving the original meaning. The Cronbach’s alpha calculated in the aforementioned pilot study was 0.72 for each of the two dimensions, indicating acceptable internal consistency.

### 2.3. Ethical Considerations

This study received formal approval from the Research Ethics Committee of the Master’s Program in Health Services Management at Universidad César Vallejo, ensuring that all ethical guidelines and research standards were thoroughly followed. Prior to data collection, each participant was provided with a detailed informed consent form outlining the purpose of this study, procedures involved, potential risks and benefits, and their rights as participants, including the right to withdraw at any time without penalty. Only individuals who voluntarily agreed and signed the informed consent form were included in the study, thus upholding the principles of autonomy, confidentiality, and respect throughout the research process.

In addition, this research was conducted in accordance with the ethical principles established in the Declaration of Helsinki, which emphasizes the importance of protecting the health, dignity, integrity, and privacy of all research participants. This study adhered to these international standards by ensuring voluntary participation, minimizing potential harm, and safeguarding participants’ well-being at all stages of the research process.

### 2.4. Data Analysis

Subsequently, the collected data were analyzed using IBM SPSS Statistics (version 25). Descriptive statistics were conducted, presenting sociodemographic data of the participants in the results section, along with measuring the variable of Spiritual Perspective and its dimensions: Spiritual Practices and Spiritual Beliefs. Given the descriptive and exploratory nature of this research, we used descriptive statistics and confirmatory factor analysis. Our aim was not to test hypotheses but rather to offer an initial understanding of spiritual perspectives in this specific patient population. Future studies may apply hypothesis-driven models or inferential analyses to further examine these relationships.

## 3. Results

### 3.1. Sociodemographic Characteristics of the Hospitalized Cancer Patients in the Study

According to Table 2, in terms of marital status, 51.09% of the respondents identified as “Single”, while 29.93% reported being “Cohabitant”, and 18.98% stated they were “Married”. Regarding occupation, the majority of respondents (52.55%) identified their occupation as “Homemaker”, followed by “Employee” at 41.61%. A smaller percentage (5.84%) identified as “Student”. Concerning education level, the highest proportion of respondents (67.88%) reported having a “High school” education, while 31.39% indicated possessing a “Superior” education level. Only a small fraction (0.73%) reported having a “Primary” education level. In terms of age, among the surveyed population, 63.50% fell into the category of “Older adult”, while 36.50% identified as “Young adult”.

The predominance of single individuals (51.09%) and homemakers (52.55%) suggests a potentially significant overlap between marital and occupational statuses, which could influence their perspectives on the study variables. The high proportion of respondents with a high school education (67.88%) reflects a population with foundational education, which may shape their understanding of and engagement with spiritual care and Spiritual Perspective. The age distribution, with the majority being older adults (63.50%), aligns with the general demographic trends often observed in oncology patient populations and underscores the importance of tailoring care approaches to meet the unique needs of this age group. Together, these characteristics offer a comprehensive profile of the study population, providing context for interpreting the findings and guiding the development of targeted interventions.

### 3.2. Level of Spiritual Perspective Based on Pamela Reed’s SPS in Hospitalized Cancer Patients

Table 3 shows the level of Spiritual Perspective (and its dimensions) based on Pamela Reed’s SPS in hospitalized cancer patients

#### 3.2.1. Spiritual Perspective

As seen in Table 3, findings on Spiritual Perspective reveal a noteworthy concentration of respondents within the “Middle” level (88.32%), indicating a prevalent moderate sense of connection and meaning among the study population. The absence of individuals in the “Low” category suggests that even at the lowest levels, the respondents maintain some degree of spiritual or existential engagement. Meanwhile, the smaller proportion of participants in the “High” category (11.68%) reflects a more profound sense of purpose and interconnectedness, which might be influenced by personal or contextual factors, such as support networks or spiritual practices.

#### 3.2.2. Dimension 1: Spiritual Practices

Once more, most respondents fall into the “Middle” level in terms of engagement in spiritual practices, accounting for 54.74% of the total. The “High” level is slightly larger, comprising 45.26% of respondents. Like the overall Spiritual Perspective level, no respondents are classified as having “Low” engagement in spiritual practices.

This suggests that all participants incorporate spiritual practices to some degree in their lives. The significant representation in the “High” category reflects a notable commitment to activities such as prayer, meditation, or reading spiritual materials, which may play a critical role in their coping mechanisms and emotional well-being. The findings underscore the relevance of spiritual practices in the holistic care of oncology patients and suggest an opportunity for healthcare providers to encourage and support these practices as part of patient-centered care.

#### 3.2.3. Dimension 2: Spiritual Beliefs

In this dimension, the “High” level has the largest proportion of respondents, comprising 59.12%. The “Middle” level represents 40.88% of the total. Once again, no respondents are categorized as having “Low” spiritual beliefs.

The absence of respondents in the “Low” category underscores the pervasive presence of spiritual beliefs among the study population. The predominance of “High” spiritual beliefs suggests a deep sense of connection to higher powers or values, which may serve as a vital source of hope, resilience, and meaning for oncology patients. These findings highlight the importance of acknowledging and integrating patients’ spiritual beliefs into holistic care plans, as they can significantly influence coping mechanisms and overall well-being.

### 3.3. Confirmatory Factor Analysis Results

The measurement model was evaluated using confirmatory factor analysis (CFA) to examine the hypothesized two-factor structure. The model specified two correlated latent factors: Factor 1 comprised Items 1 through 4, while Factor 2 included Items 5 through 10. The analysis was conducted using robust maximum likelihood estimation in lavaan (Version 0.6–16), with standard errors computed via bootstrap resampling (1000 iterations) to ensure stable parameter estimates given the sample size of 137 participants.

#### 3.3.1. Model Fit Assessment

The model demonstrated good fit to the observed data, as evidenced by multiple fit indices (see Table 4). While the chi-square test was statistically significant (χ^2^ = 48.17, df = 34, *p* = 0.047), this is commonly observed with sample sizes exceeding 100 and should be interpreted in conjunction with other fit indices. A comparative fit index (CFI) of 0.96 and a Tucker–Lewis index (TLI) of 0.94 both exceeded the recommended threshold of 0.95, indicating excellent relative fit. The root mean square error of approximation (RMSEA) of 0.06 (90% CI [0.03, 0.08]) and standardized root mean square residual (SRMR) of 0.04 both fell below their respective cutoffs of 0.08, suggesting good absolute and approximate fit.

#### 3.3.2. Factor Loadings and Reliability

All standardized factor loadings were statistically significant (*p* < 0.001) and exceeded 0.60, demonstrating strong relationships between items and their respective latent factors (Table 5). For Factor 1 (Spiritual practices), loadings ranged from 0.67 (Item 2) to 0.79 (Item 4), while Factor 2 (Spiritual beliefs) loadings ranged from 0.64 (Item 5) to 0.73 (Item 8). The composite reliability estimates of 0.83 for Factor 1 and 0.85 for Factor 2 exceeded the recommended threshold of 0.70, indicating good internal consistency. Average variance extracted (AVE) values were 0.55 for Factor 1 and 0.50 for Factor 2, approaching or meeting the 0.50 benchmark for adequate convergent validity.

#### 3.3.3. Factor Correlations and Discriminant Validity

The two latent factors showed a moderate positive correlation (r = 0.42, *p* < 0.001, 95% CI [0.30, 0.53]), suggesting they measure related but distinct constructs (Table 6). Discriminant validity was further supported by comparing the square of this correlation (0.18) to the AVE values for each factor (0.55 and 0.50), following the Fornell–Larcker criterion. The shared variance between factors (18%) was substantially less than the average variance explained by each factor’s indicators (50–55%).

#### 3.3.4. Internal Consistency

Cronbach’s alpha coefficients provided additional evidence of scale reliability, with α = 0.82 (95% CI [0.77, 0.86]) for Factor 1 and α = 0.85 (95% CI [0.81, 0.88]) for Factor 2. These estimates were consistent with the composite reliability values from the CFA, suggesting robust internal consistency across both estimation methods.

#### 3.3.5. Discussion of CFA Findings

The confirmatory factor analysis provided strong support for the hypothesized two-factor structure. All items loaded significantly on their intended factors, with standardized coefficients exceeding 0.60, demonstrating adequate to strong indicator reliability. The model fit indices collectively suggested a good fit to the data, despite the marginally significant chi-square test, which is sensitive to sample size. The moderate correlation between factors indicates they measure related but distinct aspects of the construct domain, supporting their treatment as separate but conceptually linked dimensions.

The reliability estimates exceeded recommended thresholds for both factors, suggesting the scales produce consistent measurement. While the AVE for Factor 2 (0.50) was at the lower bound of acceptability, this is not uncommon for newer scales and may reflect the breadth of content covered by its six items. Researchers using these measures should be aware that the moderate factor correlation suggests some shared variance between constructs, which may warrant consideration in analytic planning.

### 3.4. Perception of Spiritual Perspective According to Pamela Reed’s Perspective

As seen in Table 7, in terms of spiritual practices, there exists a spectrum in how frequently individuals engage with spiritual topics during conversations with family and friends, share their spiritual beliefs, delve into spiritual literature, and engage in private prayer or meditation. Most people report discussing these topics occasionally, with the frequency gradually increasing, particularly when sharing personal joys and struggles, and reading spiritual literature typically occurring about once a week. A minority also indicates engaging in these practices daily, suggesting a deeper level of commitment.

Table 6 provides an insightful breakdown of Spiritual Perspective perception based on Pamela Reed’s theoretical framework. The results indicate that a significant proportion of respondents engage in spiritual practices at least once a month, particularly in sharing spiritual experiences and reading books on the subject. A notable percentage also engage in private prayer or meditation on a daily or weekly basis. These findings suggest that spirituality plays a meaningful role in the participants’ lives, reinforcing the importance of Spiritual Perspective as a factor in personal well-being and coping mechanisms.

Additionally, the responses to spiritual beliefs highlight a complex relationship between faith and daily decision-making. While forgiveness and spirituality as guiding principles receive strong agreement from participants, the perception of feeling close to a higher power varies, with a considerable percentage leaning toward neutrality or disagreement. This divergence suggests that while individuals may identify spirituality as an important aspect of life, the intensity of their spiritual experiences and convictions may differ. The variance in agreement levels highlights the need for a more nuanced approach to understanding spirituality’s role in everyday life.

From an analytical perspective, the data could benefit from deeper statistical examination, such as correlation analysis between spiritual practices and beliefs. Identifying significant relationships between frequent spiritual activities and their impact on decision-making or coping strategies could enhance the interpretative value of the findings. Additionally, incorporating qualitative insights, such as open-ended responses on spiritual experiences, could provide a richer understanding of how individuals conceptualize and apply Spiritual Perspectives in their personal and social contexts.

**Figure 1:**
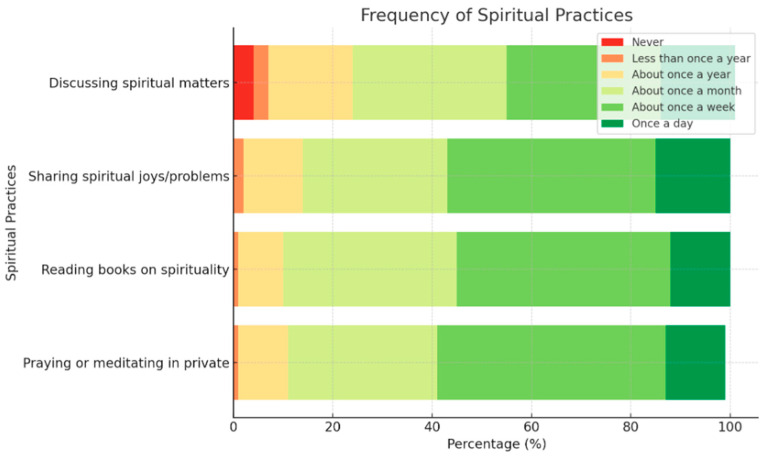
Stacked bar chart visualizing the frequency of spiritual practices among respondents.

Also, Figure 1 presents a stacked bar chart visualizing the frequency of spiritual practices among respondents. It shows how often people engage in discussing spiritual matters, sharing joys/problems, reading spiritual books, and private prayer/meditation. The majority practice these activities at least once a month, with a significant portion engaging weekly or daily.

**Figure 2:**
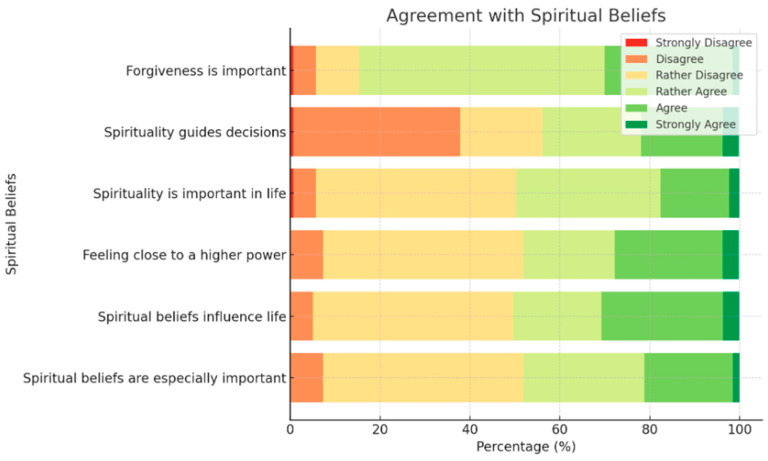
Stacked bar chart showing agreement levels with spiritual beliefs.

Also, Figure 2 presents a stacked bar chart showing agreement levels with various spiritual beliefs. It highlights how respondents perceive forgiveness, spirituality’s role in decision-making, and their connection to a higher power.

## 4. Discussion

### 4.1. Overview of Key Findings

This study represents one of the first quantitative assessments of Spiritual Perspective using Pamela Reed’s theoretical framework in a Peruvian oncology context. By applying a culturally adapted version of the Spiritual Perspective Scale, this research provides original data that can guide spiritual care practices in Latin American hospitals and contributes to the global understanding of how spiritual beliefs and practices manifest in diverse cultural settings. The findings revealed that 88.32% of hospitalized oncology patients experienced a moderate level of Spiritual Perspective, while 11.68% reached high levels and none fell into the low category. These results suggest that while most patients maintain spiritual awareness and connection, they may not fully achieve the deeper forms of meaning-making, interpersonal connection, and personal growth that define a high Spiritual Perspective. The absence of low-level scores reinforces the relevance of spirituality during illness, as noted by Silva et al. [52], highlighting its importance as a coping resource for individuals facing life-threatening conditions.

### 4.2. Interpreting Spiritual Perspective Through Reed’s Theory

According to Pamela Reed [35,37], Spiritual Perspective represents the expansion of personal boundaries in four key directions: inward (toward self-awareness), outward (toward others), temporally (connecting past, present, and future), and transpersonally (toward a higher purpose or spiritual being). In this study, moderate levels may indicate that patients are engaging in some of these dimensions but are not fully supported in others. For example, transpersonal and inward reflections might be present, while outward connections or spiritual expression could be limited by institutional or emotional constraints.

Illness often provokes existential reflection and intensifies spiritual needs [2,52]. However, when these needs are not sufficiently addressed in clinical settings, patients may experience spiritual discomfort, which can limit their ability to access the deeper healing potential of Spiritual Perspectives. This underscores the importance of creating environments where spiritual connection is acknowledged, respected, and encouraged, particularly in oncology units where vulnerability is heightened.

These results are comparable to findings in European and North American settings where moderate levels of Spiritual Perspective are commonly observed in hospitalized populations [38,53]. However, unlike some Western cohorts, the Peruvian sample reported more pronounced alignment with traditional religious practices, reflecting cultural differences in the expression of spirituality during illness.

### 4.3. Discrepancy Between Spiritual Beliefs and Practices

A significant finding was the contrast between the high level of spiritual beliefs (59.12%) and the lower level of spiritual practices (45.26%). Most patients agreed that forgiveness, inner peace, and closeness to a higher power were essential, but fewer reported engaging in daily or weekly practices such as prayer, meditation, or reading spiritual literature. This discrepancy aligns with prior studies that emphasize how institutional barriers, fatigue, lack of privacy, and unfamiliarity with available spiritual resources can inhibit spiritual practice [2,54].

This difference may also indicate spiritual unmet needs. While beliefs are sustained internally, active practice often requires space, energy, or encouragement—elements that may be lacking during hospitalization. As Reed’s theory suggests, the potential for Spiritual Perspective exists inherently but is shaped and strengthened through relational, reflective, and environmental factors [35,37]. This highlights the need to integrate simple yet meaningful spiritual practices into daily care routines.

### 4.4. Sociodemographic Patterns and Spiritual Coping

The predominance of older adults, single individuals, and homemakers with high school education suggests a specific sociocultural profile in which spirituality may serve as an emotional anchor. Individuals in these groups may rely more heavily on internal belief systems and religious values when formal social roles or economic resources are limited. These results are consistent with prior research in Peru and Latin America that identifies spirituality as a protective factor in cancer patients’ coping processes [50,55].

Moreover, aging populations tend to seek greater existential meaning and demonstrate increased spiritual awareness, particularly in contexts of suffering and illness [56,57]. These findings suggest that the moderate-to-high spiritual beliefs observed in this population reflect both cultural traditions and age-related transformations in meaning-making processes.

### 4.5. Confirmatory Factor Analysis Supports Two-Factor Structure with Strong Reliability and Validity

The confirmatory factor analysis provided strong evidence for the hypothesized two-factor structure, with all items loading significantly on their intended factors and demonstrating good reliability. The model fit indices (CFI = 0.96, RMSEA = 0.06) supported the proposed structure. These results validate the use of this measurement model for assessing these latent variables in subsequent analyses.

The findings suggest that Items 1–4 and Items 5–10 form coherent and psychometrically sound scales, though the somewhat lower AVE for Factor 2 (0.50) may warrant attention in future refinements. While the sample size (N = 137) was adequate for the analysis, replication in larger and more diverse samples would strengthen confidence in the factor structure. Overall, these results support the theoretical distinction between the two constructs while providing a robust foundation for their operationalization in future research.

### 4.6. Role of Support Networks in Promoting Spiritual Perspective

Support networks emerged in the study as crucial facilitators of spiritual well-being. Patients who experience emotional connection with family, friends, or healthcare professionals may find it easier to engage in spiritual reflection and practice. Previous studies show that emotional and social support improve quality of life in cancer patients and buffer the emotional burden of illness [58,59].

Healthcare teams should therefore view support networks not only as social resources but also as spiritual mediators. Encouraging patient–family dialogue, peer support groups, or interaction with chaplains and religious volunteers can enhance patients’ sense of connection and promote higher levels of Spiritual Perspective.

### 4.7. Implications for Nursing Practice and Holistic Care

The findings point to the urgent need for training nurses and care providers to recognize and support patients’ spiritual needs. Spiritual assessments should be integrated into nursing protocols alongside physical and psychological evaluations. Tools like spiritual history forms, reflection journals, or guided meditation programs can help bridge the gap between belief and practice.

Pamela Reed’s theory highlights the dynamic and ongoing nature of the Spiritual Perspective [35,37]. Healthcare professionals should approach spirituality not as a static belief but as a lived, evolving experience that can be nurtured through compassionate presence, meaningful rituals, and authentic communication. These strategies align with global recommendations for delivering holistic, person-centered cancer care [30,45,46].

### 4.8. Limitations and Future Research

This study has several limitations. Its cross-sectional design prevents the analysis of changes in spirituality over time or during different stages of illness. The use of self-report questionnaires introduces subjectivity and social desirability bias, which may affect how participants respond to spiritual items. Moreover, spirituality is influenced by culture, gender, religious tradition, and personal history (factors that should be explored further using mixed methods).

Another limitation of this study is the absence of clinical data such as cancer stage, diagnosis type, or religiosity level, which may influence patients’ spiritual engagement. Future research should incorporate these variables to allow for a more nuanced understanding of the factors associated with the Spiritual Perspective.

This study primarily used descriptive statistics and confirmatory factor analysis. While informative, more advanced statistical modeling (such as regression analysis) could offer deeper insight into the variables influencing spiritual beliefs and practices. Future studies should explore these relationships using inferential models.

Additionally, potential biases such as social desirability may have influenced participants to overreport spiritual engagement. The absence of a control for confounding variables—such as religious affiliation, disease severity, or psychosocial support—may also limit the interpretation of results. Future studies should control these variables using multivariate models to refine the findings.

Future research could also incorporate longitudinal designs to examine how spiritual practices evolve throughout treatment and recovery. Qualitative interviews could also provide richer insights into how patients interpret and experience the Spiritual Perspective, especially in diverse religious and cultural groups. In addition, it would be valuable to compare oncology patients with other chronic disease populations to explore the broader applicability of Reed’s model.

## 5. Conclusions

Measuring the variable of Spiritual Perspective perception from Pamela Reed’s perspective in hospitalized oncology patients holds significant promise for both patients and nursing professionals alike. By deepening nursing professionals’ understanding, this measurement can lead to enhanced experiences for hospitalized oncology patients, addressing not only the physical aspects of the disease but also the emotional and spiritual dimensions associated with Spiritual Perspectives. This holistic approach can result in more patient-centered care.

Pamela Reed’s Spiritual Perspective theory emphasizes patient-centered care, urging nursing professionals to focus on individual patient needs and deliver care that is personalized and meaningful. By identifying specific strategies to support patients’ Spiritual Perspective, nursing professionals can positively impact their overall well-being and foster a sense of hope, thereby promoting a higher quality of life despite the challenges posed by illness.

Nevertheless, it is essential to approach such studies with ethical sensitivity, recognizing the delicate nature of addressing spirituality and Spiritual Perspective in a healthcare setting. Methodological limitations and the diversity of beliefs and experiences among oncology patients must also be carefully considered. Ultimately, research of this nature enriches nursing practice and improves care by honoring the individual needs and desires of these patients.

Therefore, it is recommended that hospitals implement training programs in spiritual care, provide dedicated spaces for prayer or meditation, and encourage the integration of spiritual assessments into routine oncology care. By operationalizing these findings, nursing professionals can better support patients’ inner strengths and coping mechanisms, ultimately contributing to more humanized and effective healthcare services.

## 6. Implications for Healthcare Management

Given that many patients engage in spiritual practices such as prayer, meditation, and discussions on faith, integrating holistic patient care into hospital policies becomes essential. Hospitals could enhance patient satisfaction by providing interfaith chaplaincy services, dedicated prayer and meditation spaces, and staff training on recognizing and addressing patients’ spiritual needs. Acknowledging spirituality in healthcare may foster a sense of comfort and hope, ultimately contributing to better emotional and physical recovery outcomes.

Beyond patient care, these insights have important implications for healthcare workers’ well-being and decision-making. While many professionals value spirituality, they may not always have time to engage in it actively. The stressful nature of hospital work often leads to burnout, making it crucial for management to implement mindfulness programs, peer support groups, and ethical decision-making training rooted in spiritual perspectives. Encouraging hospital staff to reflect on their own values and spiritual well-being could help them handle emotional distress, particularly in high-pressure scenarios such as palliative care and end-of-life decisions.

Additionally, effective communication between healthcare providers and patients is essential, especially when addressing sensitive topics such as illness, suffering, and recovery. Hospitals could provide counseling services that integrate both psychological and spiritual support. Furthermore, mental health professionals and social workers could work alongside spiritual leaders to develop programs that help both patients and caregivers cope with stress, grief, and uncertainty. By fostering a hospital culture that recognizes the role of spirituality in healing, healthcare management can contribute to a more empathetic and humanized healthcare system, ultimately improving both patient and staff experiences.

## Figures and Tables

**Table 1:** Spiritual Perspective Scale (SPS).

Item	Spiritual Practices
1	When talking with your family or friends, how often do you discuss spiritual matters?
2	How often do you share with others the struggles and joys of living according to your spiritual beliefs?
3	How frequently do you read books about spiritual topics?
4	How often do you pray or meditate in private?
	Spiritual Beliefs
5	Is forgiveness an important part of your spiritual life?
6	Do you see spirituality as a guide for making decisions in your daily life?
7	Are your spiritual beliefs an important part of your life?
8	Have you recently felt very close to God, or to a “higher power,” during significant moments in your daily life?
9	Do your spiritual beliefs influence your daily life?
10	Are your spiritual beliefs especially important to you?

**Table 2:** Sociodemographic characteristics of the hospitalized cancer patients in this study.

Marital Status			Occupation			Level of Education			Age		
Single	70	51.09%	Employee	57	41.61%	Primary	1	0.73%	Older adult	87	63.50%
Married	26	18.98%	At home	72	52.55%	High school	93	67.88%	Young adult	50	36.50%
Cohabitant	41	29.93%	Student	8	5.84%	Superior	43	31.39%	-	-	-
Total	137	100%	Total	137	100%	Total	137	100%	Total	137	100%

**Table 3:** Level of Spiritual Perspective based on Pamela Reed’s SPS in hospitalized cancer patients.

Level	Variable		Dimension		Dimension	
	Spiritual Perspective		Spiritual Practices		Spiritual Beliefs	
	*n*	%	*n*	%	*n*	%
Low	0	0.00%	0	0.00%	0	0.00%
Middle	121	88.32%	75	54.74%	56	40.88%
High	16	11.68%	62	45.26%	81	59.12%
Total	137	100.00%	137	100.00%	137	100.00%

**Table 4:** Confirmatory Factor Analysis Model Fit Indices.

t Index	Value	Recommended Threshold	Interpretation
χ^2^ (df)	48.17 (34)	*p* > 0.05	Marginal absolute fit
CFI	0.96	≥0.95	Excellent relative fit
TLI	0.94	≥0.95	Good relative fit
RMSEA	0.06	≤0.08	Good approximate fit
90% CI	[0.03, 0.08]		
SRMR	0.04	≤0.08	Excellent residual fit

**Table 5:** Standardized Factor Loadings and Reliability Estimates.

Item	Factor Loading	Standard Error	*p*-Value	Item Reliability (R^2^)
Factor 1				
1	0.74	0.05	<0.001	0.55
2	0.67	0.06	<0.001	0.45
3	0.71	0.05	<0.001	0.5
4	0.79	0.04	<0.001	0.62
Factor 2				
5	0.64	0.06	<0.001	0.41
6	0.7	0.05	<0.001	0.49
7	0.68	0.05	<0.001	0.46
8	0.73	0.04	<0.001	0.53
9	0.69	0.05	<0.001	0.48
10	0.65	0.06	<0.001	0.42

Note: Composite reliability (Factor 1 = 0.83, Factor 2 = 0.85); average variance extracted (Factor 1 = 0.55, Factor 2 = 0.50).

**Table 6:** Factor Correlation Matrix with Confidence Intervals.

	Factor 1	Factor 2
Factor 1	1	
Factor 2	0.42 ***	1

Note: *** *p* < 0.001; 95% CI [0.30, 0.53].

**Table 7:** Perception of Spiritual Perspective According to Pamela Reed’s Perspective.

1. Spiritual Practices							
Spiritual Practice	Never (1)	Less than Once a Year (2)	About Once a Year (3)	About Once a Month (4)	About Once a Week (5)	Once a Day (6)	Key Insight
Discussing spiritual matters with family or friends	4%	3%	17%	31%	31%	15%	Most respondents engage in spiritual discussions at least monthly.
Sharing spiritual joys and problems	0%	2%	12%	29%	42%	15%	Majority share their spiritual experiences at least weekly.
Reading books on spirituality	0%	1%	9%	35%	43%	12%	Reading spiritual books is a common practice, with 90% reading at least yearly.
Praying or meditating in private	0%	1%	10%	30%	46%	12%	Private prayer/meditation is frequent, with 88% engaging at least monthly.
**2. Spiritual Beliefs**							
**Spiritual Belief**	**Strongly Disagree (1)**	**Disagree (2)**	**Rather Disagree (3)**	**Rather Agree (4)**	**Agree (5)**	**Strongly Agree (6)**	**Key Insight**
Forgiveness is an important part of spirituality	0.70%	5.10%	9.50%	54.70%	28.40%	1.50%	Majority (83%) believe in the importance of forgiveness.
Spirituality as a guide in daily decisions	0.70%	37.20%	18.20%	21.90%	18.20%	3.60%	Spirituality plays a role in decisions for 44%, but 37% disagree.
Spiritual beliefs are important in life	0.70%	5.10%	44.50%	32.10%	15.30%	2.20%	Importance of spirituality is mixed, with 50% in agreement.
Feeling close to a higher power	0%	7.30%	44.50%	20.40%	24.00%	3.60%	Around 48% report a connection with higher power, while 44% are unsure.
Spiritual beliefs influence daily life	0%	5.10%	44.50%	19.70%	27.00%	3.60%	50% believe spirituality affects daily life, but 44% disagree.
Spiritual beliefs are especially important	0%	7.30%	44.50%	27.00%	19.70%	1.50%	About 48% consider spirituality important, but 44% are undecided.

## Data Availability

The data that support the findings of this study are available from the corresponding author upon reasonable request.
